# Seroprevalence, Risk Factors and Molecular Identification of Bovine Leukemia Virus in Egyptian Cattle

**DOI:** 10.3390/ani11020319

**Published:** 2021-01-27

**Authors:** Abdelfattah Selim, Eman A. Manaa, Abdullah D. Alanazi, Mohamed S. Alyousif

**Affiliations:** 1 Department of Animal Medicine (Infectious Diseases), Faculty of Veterinary Medicine, Benha University, Toukh 13736, Egypt; 2 Animal and Poultry Production, Department of Animal Wealth Development, Faculty of Veterinary Medicine, Benha University, Toukh 13736, Egypt; Eman.Manaa@fvtm.bu.edu.eg; 3 Department of Biological Sciences, Faculty of Science and Humanities, Shaqra University, P.O. Box 1040, Ad-Dawadimi 11911, Saudi Arabia; aalanazi@su.edu.sa; 4 Department of Zoology, College of Science, King Saud University, P.O. Box 2455, Riyadh 11451, Saudi Arabia; myousif@ksu.edu.sa

**Keywords:** bovine leukemia virus, seroprevalence, risk-factors, phylogenetic analysis, cattle

## Abstract

**Simple Summary:**

Bovine leukemia virus (BLV) is distributed worldwide and affects dairy cattle causing significant economic losses. This study’s objective was to assess the risk factors associated with BLV infection and identify the Egyptian BLV strain’s genetic diversity. The overall seroprevalence of BLV infection in Egyptian dairy cattle was 18.2%, and the grazing cattle in the losing house system had a higher probability of getting BLV infection. The sequencing and phylogenetic analysis for one Egyptian BLV strain was performed, and the obtained results confirmed the clustering of Egyptian BLV strain into genotype-1.

**Abstract:**

Bovine leukemia virus (BLV) is distributed worldwide and affects dairy cattle causing severe economic losses. The BLV has been serologically reported in Egypt, but few studies have evaluated its associated risk factors and genetic classification. Therefore, this study assessed risk factors associated with BLV infection and identified the genetic diversity of the Egyptian strain. The study was conducted on 500 dairy cattle distributed in four Governorates located in Northern Egypt. Overall, the seroprevalence of BLV infection among Egyptian dairy cattle was 18.2%. The grazing cattle in the losing house system had higher odds for BLV seropositivity, and bad practice such as the use of a single needle or one plastic glove for more than one animal was considered a significant risk factor for BLV infection. Besides, the sequencing and phylogenetic analysis for one Egyptian BLV strain was performed, and the obtained results confirmed the clustering of Egyptian BLV strain into genotype-1. The assessment of associated risk factors for BLV infection and determination of its genetic classification are essential to implement an effective control program.

## 1. Introduction

Bovine leukemia virus (BLV) is an etiological agent of enzootic bovine leukosis (EBL), belonging to the genus of Deltaretrovirus of the family Retroviridae [1,2].

EBL is spread worldwide and is highly prevalent in North and South America, Asia, Eastern Europe, and some Middle Eastern countries [3,4,5,6]. BLV could be shed in the various secretions of infected animals such as blood, milk, semen, saliva, and nasal secretion and transmitted mainly through vertical and horizontal routes particularly via biting insects [7,8]. Horizontal transmission can occur by direct contact of mucosa or the abraded skin of susceptible animals with infected secretions. Clinically, EBL in cattle has three stages: first, the silent stage, characterized by the aleukemic form in which the animal is serologically positive without lymphocytosis; second, persistent lymphocytosis (PL) occurring in 30% of infected animals, characterized by polyclonal expansion of B cells and the majority of which harbor the BLV provirus; third, leukemia 1–10% of infected animals could develop malignant lymphosarcoma with a long period of latency of about 1–8 years [3,9].

Additionally, the distribution of EBL among dairy cattle causes severe economic losses due to prematurely culling, poor reproductive performance, decreased milk production, and the longevity of infected cattle [10,11,12]. Thus, the investigation and elimination of cattle with high viral loads are crucial in controlling the disease and sustaining animal production [13].

Serological tests are widely used for screening animals for EBL. The antibodies most readily detected in BLV-infected animals’ blood are those directed toward the virus antigens gp51 of env protein and p24 of gag protein [1]. The age of animals plays a pivotal role as the BLV sero-prevalence in cattle older than two years is almost twice as high as in younger animals, as older animals have spent a long time at risk and more likely to have become BLV-infected [14]. Several pieces of research studied the risk factors related to BLV infection in cattle, where the infection increase in animals reared in a large herd (>200) or for old cattle (Parity > 5) [15,16]. Also, direct contact with discharges of infected animals particularly in the communal pasture increases the risk of infection, while using a single needle poses a high risk for transmission of infection between susceptible animals [13,17,18].

The BLV genome comprises structural and enzymatic gag, pro, pol, and env genes, regulatory genes tax and rex, accessory genes R3 and G4. The BLV env gene encodes structural proteins that consist of gp30 transmembrane (TM) protein and gp51 surface glycoprotein (SU) [19,20]. The env gp51 glycoprotein plays an essential role in the viral life cycle, necessary for cell entry and neutralizing antibodies. Thus, the gp51 is widely used for molecular characterization and genotyping of BLV [21,22].

Previous sequence analysis studies based on gp51 region classified the BLV genome into seven distinct genotypes [23]. Subsequently, another phylogenetic study identified genotype 8 among BLV samples in Croatia [24]. Furthermore, analysis of the whole BLV genome revealed the presence of a new genotype (genotype-9) in Bolivia, and another molecular epidemiology study for BLV infection in Thailand confirmed the existence of a further genotype (genotype-10) [25,26]. Recently, the newest BLV genotype (genotype-11) was discovered in China in 2019 [20].

In Egypt, EBL has not been identified clinically among cattle, but antibodies against BLV infection have been reported serologically among dairy cattle in some Governorates in northern and upper Egypt [15,27,28]. Recently, one study investigated the genetic diversity of the BLV genome in Egypt [21].

Therefore, the present study aimed to determine the recent seroprevalence and to evaluate the associated risk factors for BLV infection. The genetic classification of BLV was also identified in this study.

## 2. Materials and Methods

### 2.1. Ethical Statement

The study was approved by the Faculty of Veterinary medicine’s ethical committee, Benha University (BUFVTM). The blood samples were collected following ethical guidelines and under the owner’s consent.

### 2.2. Study Area

**Figure 1:**
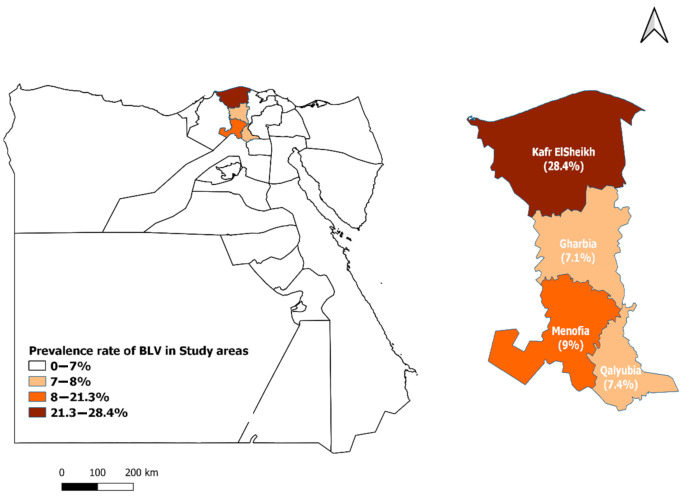
Seroprevalence rate and location of different Governorates in the study.

The study was conducted on dairy cattle, raised in four Governorates namely, Kafr ElSheikh, Menofia, Gharbia, Qalyubia geographically situated at 38°18 N to 30°56 E; 30.52° N 30.99° E; 30.867° N 31.028° E and 30°25 N to 31°13 E, Figure 1.

The selected regions’ climate has a moderate temperature in summer, but the hottest temperatures are reported only in July and August, and annual rainfalls occur usually in the winter. The selected region is an agricultural area and has large pasture suitable for grazing and raising of animals.

### 2.3. Study Design

A cross-sectional study was carried out on dairy cattle in four Governorates in Northern Egypt during 2019. The sample size was calculated by Thrusfield’s formula as follow:

$$n=\frac{Z^{2}\times P\left(1-P\right)}{d^{2}}$$

where *n* is the sample size, *P* = 0.285, *Z* = 1.96, and the precision is (*d* = 0.05). This study determined the sample size based on an expected prevalence rate of 17.7%, as previously reported by Selim et al. [15], 95% confidence interval, and 5% absolute precision.

A total of 500 blood samples was collected from dairy cattle during 2019. Cattle were categorized according to locality (Kafr ElSheikh, Menofia, Gharbia, and Qalyubia) and housing conditions (loose housing and tie housing). Also, information about animal management was acquired from veterinarian or farmers, such as grazing, use of plastic gloves for rectal examination, and change of needles for treatment and vaccination.

Blood samples (5 mL) were obtained from the jugular vein with and without coagulant. The clotted samples were centrifuged at 3000× *g* for 10 min for separation of serum and kept at −20 °C for serological examination.

### 2.4. Serological Examination Using ELISA

According to the manufacturer’s instructions, the antibodies against BLV infection were detected using IDEXX Leukosis Serum Screening Ab Test (IDEXX laboratories, Westbrook, ME, USA). The optical density was measured at 450 nm using an ELISA microplate reader. The results were expressed as a sample to a positive percentage (S/P%), where S/P% ≥ 60% was considered positive for BLV antibodies.

### 2.5. Extraction of Genomic DNA

The DNA was extracted from the buffy coat separated from all the blood using QIAamp DNA Mini Kit (Qiagen Ltd., West Sussex, UK) according to the manufacturer’s instructions. Afterward, the concentration of genomic DNA was measured by a nano-spectrophotometer and stored at −20 °C until PCR examination.

### 2.6. Detection of BLV Proviral DNA by PCR

The BLV env gene was amplified by PCR using specific pairs of primer that were previously evaluated by Asfaw et al. [29].

The forward primer was env gene env-F (5′-TCTGTGCCAAGTCTCCCAGATA-3′), and the reverse primer was env-R (5′-AACAACAACCTCTGGGAAGGGT-3′) to amplify the gp51 encoding region. The PCR assay was performed in 25 µL volume, comprised of 12.5 µL Dream Taq Green PCR master mix (2×) (Thermo Scientific, Schwerte, Germany), 1 µL from each primer (20 pmol/), 5.5 µL nuclease-free water, and 5 µL DNA template. Amplification was carried out by initial denaturation at 95 °C for 5 min followed by 40 amplification cycles of denaturation at 95 °C for 30 s, annealing at 60 °C for 30 s, and extension at 72 °C for 1 min, then a final extension of 72 °C for 5 min. The PCR products were electrophoresed on 1.5% agarose gel, stained by ethidium bromide, and visualized by UV.

### 2.7. Nucleotide Sequencing and Phylogenetic Analysis

According to the manufacturer’s instructions, the PCR amplicon was extracted and purified from the gels using QIAquick Gel Extraction Kit (QIAGEN, Hilden, Germany).

Afterward, the sequence env gene was performed in both directions using the same primers of PCR assay and ABI PRISM^®^ BigDye TM Terminators v3.1 Cycle Sequencing Kit (Applied Biosystems, Waltham, MA, USA). The sequences of forwarding and reverse primers were edited by the BioEdit program and assembled into consensus sequences submitted to GenBank under accession number (LC583749). This study’s sequence was aligned with other reference databases in BLAST using MEGA7 program, and finally, phylogenetic analysis was constructed based on nucleotide sequences by the neighbor-joining tree method with 1000 bootstrap replicates using the MEGA7 program.

### 2.8. Statistical Analysis

Data of the seroprevalence study was analyzed by chi-square test using SPSS V24 (IBM, USA). A *p*-value of <0.05 is considered significant, and logistic regression analysis was performed to evaluate each variable’s association and the prevalence of BLV infection.

## 3. Results

The overall seroprevalence of BLV among dairy cattle was 18.2%. The seroprevalence of BLV significantly varied between different localities under the study. The highest regional seroprevalence was observed in Kafr ElSheikh Governorate (28.4%) in comparison to other areas.

The univariant logistic regression test was performed on protentional risk factors for BLV seropositivity. The results revealed that the number of seropositive cattle increased significantly among gazing animals (26.3%, 95% CI: 17.8–36.8), raised under loose housing conditions (31.5%, 95% CI: 24.1–39.9). Some bad practices were significantly associated with BLV prevalence, such as an unchanged plastic glove during rectal examination (36.6%, 95% CI 29.3–44.6) and using a single needle for multiple animals during periodical vaccination (24.6%, 95% CI: 18–2.6), Table 1.

All examined variables were significant in the univariant analyses and fitted for a multivariant logistic regression model to determine their effect on BLV infection prevalence. The results showed that losing housing (OR = 2.9, 95% CI: 1.83–4.74), grazing (OR = 1.7, 95% CI: 1.01–3.11), using one plastic glove in the rectal examination for more than one animal (OR = 5.1, 95% CI: 3.12–8.15), and using of a single needle for vaccination of several animals (OR = 1.1, 95% CI: 0.62–1.83) were significantly associated with BLV seropositivity, Table 2.

**Figure 2:**
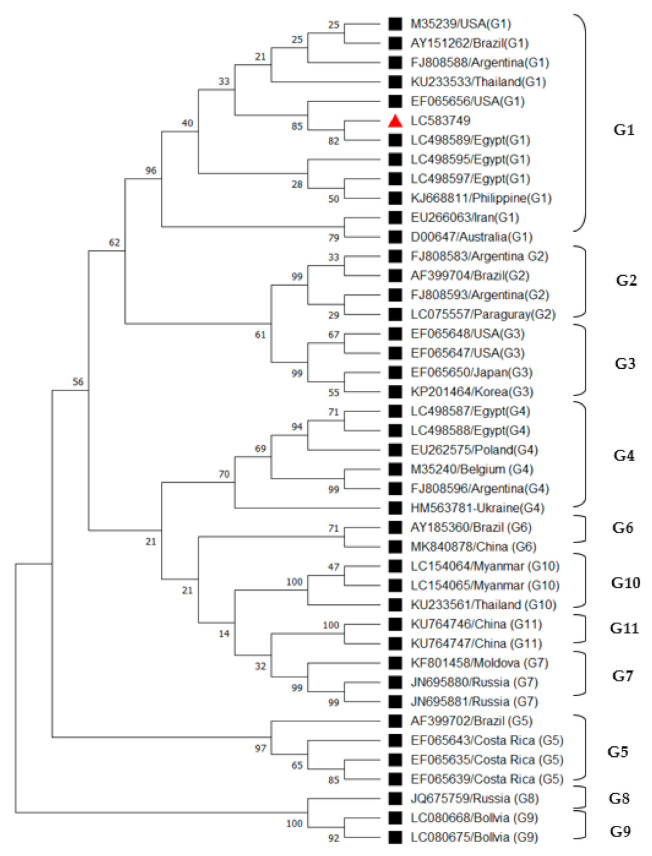
Phylogenetic tree based on env gene for Egyptian Bovine leukemia virus (BLV) and other reference strain in the database.

A neighbor-joining phylogenetic analysis based on env gene encoding gp51 sequence was constructed for the obtained sequence in this study with 42 reference strains representing BLV genotypes G1 to G11 from different countries. The phylogenetic tree designated the Egyptian strain and other reference strains in eleven distinct genotypes (G1–G11) and confirmed that the Egyptian BLV strain (LC583749) belongs to genotype 1, Figure 2.

Moreover, the Egyptian BLV strain (LC583749) was closely related to another Egyptian strain (LC498589) and USA strain (EF065656). The present study’s Egyptian BLV strain was grouped with isolates from Egypt, USA, Brazil, Argentina, Thailand, Iran, Australia, and the Philippines and clustered together to form genotype 1, Figure 2.

## 4. Discussion

EBL is a chronic infectious disease, affecting mainly dairy cattle, causing severe economic losses due to reduced milk production, premature culling, and reduction in the median age of cattle [29,30]. The disease is globally distributed and recently was reported in several Governorates in north or upper Egypt [15]. Therefore, it is essential to identify and assess the risk factors associated with the prevalence of BLV infection in dairy cattle to enable disease control and determine the Egyptian BLV stain’s genetic classification.

Overall, the antibodies against BLV infection were detected in 91 out of 500 (18.2%) dairy cattle. The seroprevalence rate varied significantly between Governorates under the study, it was highest in Kafr ElSheikh Governorate (28.4%), and the lowest rate was reported in Gharbia Governorate (7.1%). Our study found a significant association between BLV infection and locality; this may be due to different ecological, geographical factors, rearing systems, and control measures [8,15,31,32,33,34].

Moreover, the Kafr ElSheikh Governorate is famous for rice agriculture and has many swamp water areas suitable for insect multiplication. Consequently, we believe that the high insect density plays a significant role in the horizontal transmission of BLV infection among cattle in the highly prevalent areas [16,35].

Overall, the reported seroprevalence rate comes in accordance with other previous rates reported in some countries: 21.5% in Egypt, 17.7% in the Nile Delta of Egypt, 22.1% in Iran [21,27,35].

On the contrary, our reported rate was lower than other previous prevalence rates; 32.8% in Iran [22], 30% in China [20], 11–100% in Thailand [25], 62% in Colombia [36], and 81.8% in Taiwan [17]. In addition, less than 6% of cattle got BLV infection such as in Mongolia, 3.8% [19], Taiwan 5.8%, and Cambodia 5.3%. On comparing with other Middle East findings, the reported rate was higher than other rates reported in Israel 5% [37] and Saudi Arabia 20.2% [38].

In Egypt, the importation of unscreened heifer for BLV infection or using frozen semen contaminated with BLV for artificial insemination has a significant role in the prevalence of BLV among Egyptian cattle [15,27].

Interestingly, the absence of a control program, periodical surveillance for BLV infection among dairy cattle, periodical markets, and random movement of animals between Governorates could play an essential role in transmitting the disease [21,39,40,41].

Concerning house condition, BLV infection’s seroprevalence was significantly increased among cattle kept in the loose housing system, 2.9 times that of the tie housing system. This finding was consistent with the findings of Kobayashi et al. [8]. The susceptible cattle could have been infected with BLV by contaminating mucous membrane or abraded skin by infected discharges. Therefore, the possibility of direct contact between infected and uninfected cattle that are reared in the loose house system is more common, especially during feeding and watering, which increases the chance of horizontal transmission [17].

Moreover, BLV infection seroprevalence was significantly higher in grazing animals at risk of getting BLV infection, 1.7 times more than non-grazing animals. This may be attributed to free contact between uninfected and infected cattle from different communal grazing sources [8,31,33,42]. Also, detection of BLV in saliva, milk, and nasal secretion of infected animals provides a statement of bad handling or absence of regular cleaning or disinfection and lack of routine husbandry, for cattle in the loose housing system increase the probability of BLV transmission [6,43].

Interestingly, veterinarians’ or farmers’ unhygienic practice, such as using one needle for treatment or vaccination of several animals and not changing the plastic glove during rectal palpation, has been strongly associated with the prevalence of BLV infection. The present findings agree with previous studies [8,15,27] which confirmed that bad management could play an essential role in the horizontal transmission of BLV infection [35].

Analysis of partial BLV env gene sequence showed that BLV clustered into 11 genotypes, and the Egyptian strain was clustered into genotype-1. Genotype-1 has been detected worldwide in most continents like Asia, America, Europe, and Australia. Furthermore, genotype-1 covered most areas in Europe and America. The present study confirmed the previous evidence of Egyptian BLV isolate belonging to genotype-1 or genotype-4 [21]. We believe that BLV infection was introduced to Egyptian dairy cattle by importing unscreened animals from endemic countries or using contaminated frozen semen [44].

## 5. Conclusions

The present study confirms the presence of antibodies against BLV infection in Egyptian dairy cattle, decreasing animal production and longevity. Furthermore, the loose housing system, grazing, and bad management such as using one needle or one plastic glove during the handling of animals present a significant risk factor for the prevalence of BLV infection in cattle. Also, phylogenetic analysis of BLV env-gp51 sequence confirmed that the Egyptian BLV strain belongs to genotype-1. Hence, the assessment of associated risk factors for BLV infection among Egyptian dairy cattle and determination of its genetic classification are essential to implement an effective control program. 

## Figures and Tables

**Table 1:** Univariant logistic analysis for variables associated with seroprevalence of Bovine leukemia virus (BLV) infetion.

Variable	Total Number	Number of Positive Animals	% of Positive Animals	95%CI	Statistic
Locality					
Kafr Elsheikh	250	71	28.4	23.1–34.2	χ^2^ = 36.361, df = 3, *p* = 0.0001 *
Menofia	100	9	9	4.8–16.2	
Gharbia	70	5	7.1	3.1–15.6	
Qalyubia	80	6	7.5	3.4–15.4	
Housing condition					
Tie housing	370	50	13.5	10.4–17.3	χ^2^ = 20.99, df = 1, *p* = 0.0001 *
Loose housing	130	41	31.5	24.1–39.9	
Grazing					
No	420	70	16.6	13.4–20.5	χ^2^ = 4.146, df = 1, *p* = 0.042 *
Yes	80	21	26.3	17.8–36.8	
Plastic gloves for a rectal examination					
one glove per animal	350	36	10.3	7.5–13.9	χ^2^ = 49.085, df = 1, *p* = 0.0001 *
one glove for more than one animal	150	55	36.6	29.3–44.6	
The needle used for vaccination					
One needle for each animal	370	59	16	12.5–20	χ^2^ = 4.857, df = 1, *p* = 0.028 *
One needle for more than one animal	130	32	24.6	18–32.6	

CI: confidence interval. * The result is significant at *p* < 0.05.

**Table 2:** Multivariant logistic regression analysis for associated risk factors to BLV infection.

Variable		B	SE	OR	95% CI for OR	*p*-Value
Housing condition	Loose housing	1.081	0.242	2.9	1.83–4.74	0.0001 *
Grazing	Yes	0.576	0.286	1.7	1.01–3.11	0.04 *
Plastic gloves for a rectal examination	One glove for more than one animal	1.619	0.244	5.1	3.12–8.15	0.0001 *
The needle used for vaccination	One needle for more than one animal	0.071	0.274	1.1	0.62–1.83	0.79

B: Logistic regression coefficient; SE: Standard error; OR: Odds ratio; CI: Confidence interval; * The result is significant *p* < 0.05.

## Data Availability

The datasets used and analyzed during the current study are available from the corresponding author on reasonable request.
